# Breast cancer surgery under mechanical circulatory support in a patient with cancer therapy-related cardiac dysfunction: a case report

**DOI:** 10.1093/ehjcr/ytaf407

**Published:** 2025-08-23

**Authors:** Yoshihito Saijo, Hirotsugu Yamada, Robert Zheng, Soichiro Sasa, Masataka Sata

**Affiliations:** Cardiovascular Department, Tokushima University Hospital, 7708503 Kuramoto-cho, Tokushima 2-50-1, Japan; Cardiovascular Department, Tokushima University Hospital, 7708503 Kuramoto-cho, Tokushima 2-50-1, Japan; Cardiovascular Department, Tokushima University Hospital, 7708503 Kuramoto-cho, Tokushima 2-50-1, Japan; Department of Thoracic, Endocrine Surgery and Oncology, Tokushima University Hospital, 7708503 Kuramoto-cho, Tokushima 2-50-1, Japan; Cardiovascular Department, Tokushima University Hospital, 7708503 Kuramoto-cho, Tokushima 2-50-1, Japan

**Keywords:** Cancer therapy-related cardiac dysfunction, Heart failure, Mechanical circulatory support, Breast cancer, Case report

## Abstract

**Background:**

The incidence of cancer therapy-related cardiac dysfunction is increasing with the growing number of breast cancer patients. In particular, patients with active cancer combined with severe irreversible cardiac dysfunction present significant challenges in treatment decision-making.

**Case summary:**

A 40-year-old woman with Stage II HER-2-positive breast cancer received anthracycline followed by HER2-targeted agents. She developed severe heart failure due to cancer therapy-related cardiac dysfunction, with haemodynamic instability necessitating mechanical circulatory support using percutaneous cardiopulmonary support and Impella 5.5. Several weaning attempts of Impella 5.5 failed. Due to limitation for the duration of Impella support, right breast tumour resection and axillary lymph node dissection were performed under ongoing Impella support. The procedure was completed without surgical complications including significant bleeding. The patient was transferred to a specialized cardiovascular institution for possible left ventricular assist device evaluation.

**Discussion:**

This case report provides valuable insight into treatment decision-making in complex cases of irreversible cancer therapy-related cardiac dysfunction, where both oncologic and cardiologic considerations must be balanced under time-limited support devices.

Learning points

- Multidisciplinary decision-making is essential when managing patients with active cancer and decompensated heart failure.
- Proceeding with cancer resection during mechanical circulatory support may improve long-term prognosis by enabling subsequent eligibility for left ventricular assist device (LVAD) implantation or heart transplantation.
- Breast cancer surgery can be safely performed under Impella support without major perioperative complications, even in patients with severe cardiac dysfunction.

## Introduction

The incidence of cancer therapy-related cardiac dysfunction (CTRCD) is increasing with the growing number of breast cancer patients.^1,2^ Cancer therapy-related cardiac dysfunction elevates mortality risk due to heart failure and the potential interruption of cancer therapy, which may lead to cancer progression.^3,4^ In particular, patients with active cancer combined with severe irreversible cardiac dysfunction present significant challenges in treatment decision-making. In selected cases, cancer resection may be feasible under mechanical circulatory support (MCS), even when cardiac function is critically impaired.^5,6^ This approach may improve long-term prognosis by enabling subsequent left ventricular assist device (LVAD) implantation or heart transplantation. However, there are few reports regarding the safety of breast cancer surgery under MCS.

## Summary figure

**Figure:**
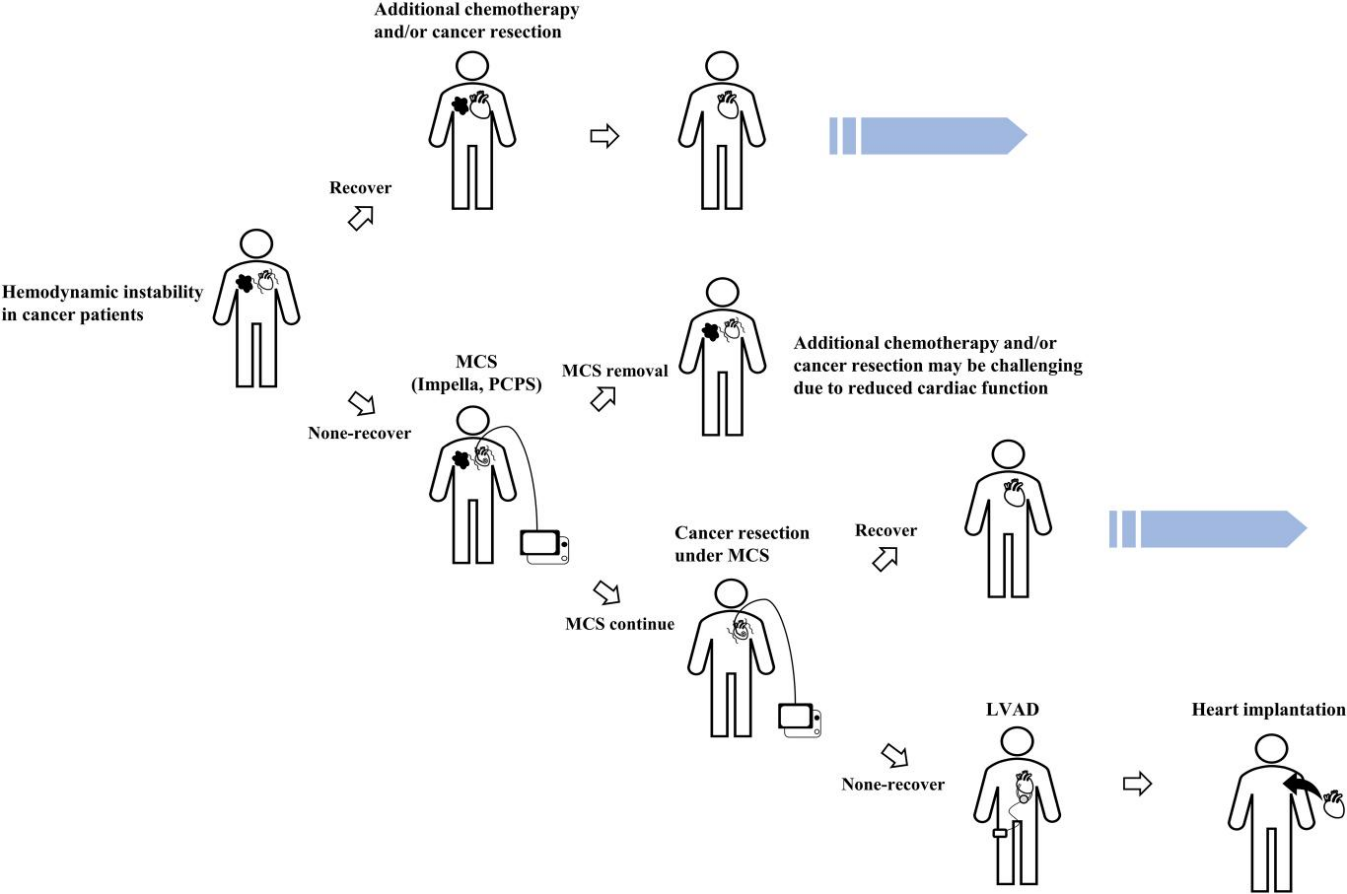
Treatment decision-making for cancer patients with severe heart failure. When cardiac recovery is insufficient, patients are considered as high-risk candidates for chemotherapy or surgery under general anaesthesia. In such cases, proceeding with cancer resection under maintaining Impella support may offer a feasible strategy to improve long-term outcomes by enabling future left ventricular assist device (LVAD) implantation or heart transplantation.

## Case presentation

**Figure 1:**
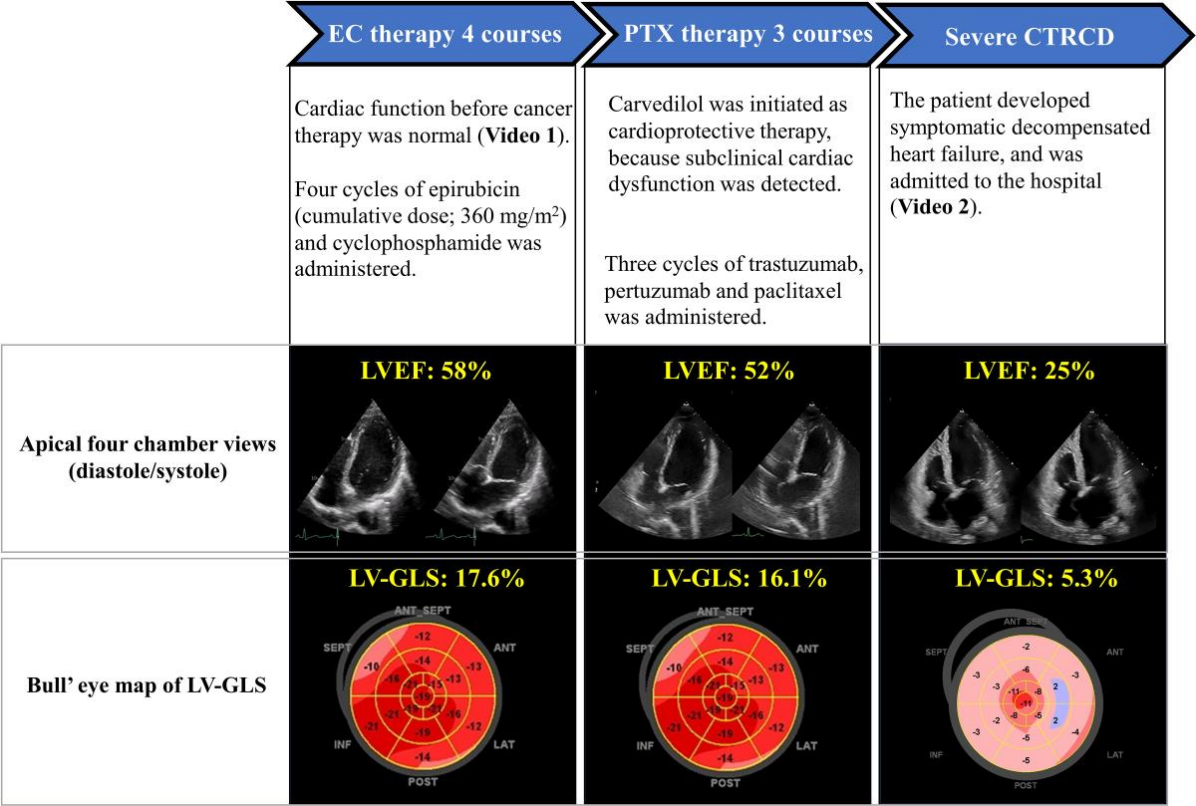
Timeline of the clinical course before admission. Cardiac function was normal prior to the initiation of cancer therapy. The patient received four cycles of epirubicin (cumulative dose, 360 mg/m^2^) and cyclophosphamide. Carvedilol was initiated as cardioprotective therapy because subclinical cardiac dysfunction was detected on echocardiography. After three cycles of pertuzumab, trastuzumab, and paclitaxel therapy, the patient developed symptomatic decompensated heart failure and was admitted to the hospital. LVEF, left ventricular ejection fraction; GLS, global longitudinal strain.

**Figure 2:**
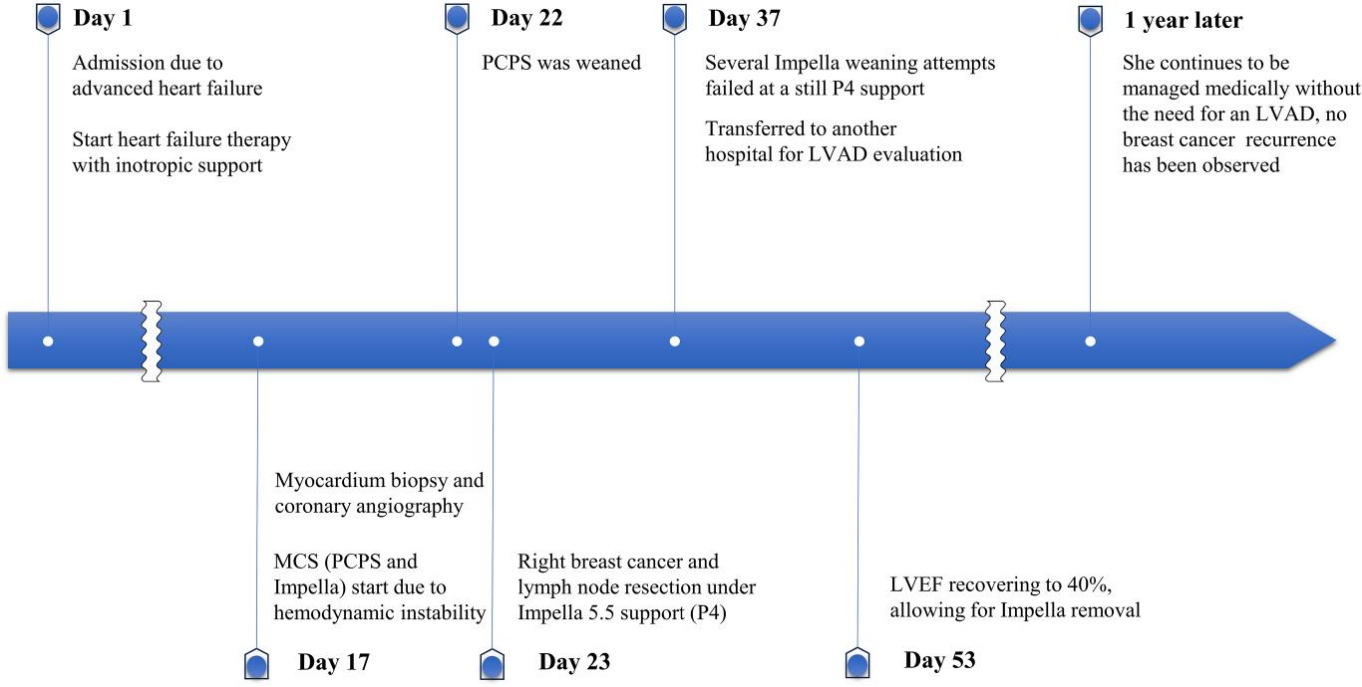
Timeline of the clinical course after admission. Timeline of clinical courses begins on the day of hospital admission. On Day 17, mechanical circulatory support was initiated due to circulation collapse. On Day 23, right breast cancer and lymph node resection were performed under Impella support (P4). Several attempts to wean from Impella were unsuccessful while still on P4 support, and the patient was transferred to another hospital for left ventricular assist device evaluation on Day 37. MCS, mechanical circulatory hospital; PCPS, percutaneous cardiopulmonary support device; LVAD, left ventricular assist device.

A 40-year-old woman was diagnosed with Stage II invasive carcinoma of the right breast, characterized by high proliferative activity, positive hormone receptor status, and HER2 overexpression. The timeline of the clinical course before admission is shown in *Figure 1*. Baseline echocardiography before chemotherapy showed a left ventricular ejection fraction (LVEF) of 58% (see Supplementary material online, *Video S1*) and a global longitudinal strain (LV-GLS) of −18%. After four cycles of epirubicin (cumulative dose, 360 mg/m^2^) and cyclophosphamide, subclinical cardiac dysfunction was detected, with LVEF reduced to 52% and LV-GLS to −16%. Carvedilol was initiated as cardioprotective therapy, and the patient was followed closely at 2-week intervals to monitor clinical status and treatment response. Due to hypotension, up-titration of carvedilol or additional cardioprotective therapy could not be administered. Following three cycles of trastuzumab, pertuzumab, and paclitaxel, she developed symptomatic decompensated heart failure with LVEF dropping to 25% (see Supplementary material online, *Video S2*), necessitating hospitalization. Despite optimal heart failure treatment, including inotropic support (dobutamine 2–5γ), circulatory collapse occurred 17 days after admission (*Figure 2*). Mechanical circulatory support was initiated using percutaneous cardiopulmonary support (PCPS) and an Impella 5.5 SmartAssist device via the right subclavian artery. Coronary angiography and cardiac biopsy were then performed. Percutaneous cardiopulmonary support was successfully discontinued on the fifth day (Day 22). However, removal of the Impella device was not feasible due to insufficient haemodynamic recovery. Endomyocardial biopsy revealed vacuolar degeneration of cardiomyocytes, myocardial disarray, and fibrosis, indicating irreversible cardiac dysfunction. As a result, the treatment strategy shifted towards LVAD implantation. After a multidisciplinary discussion, taking into account the risk of cancer resection on Impella support, right breast cancer resection and axillary lymph node dissection were performed during ongoing Impella support under anticoagulation with an activated clotting time of 160–180 s (Day 23). No significant surgical complications occurred. Several Impella weaning attempts failed at P4 support due to low cardiac output. On Day 37, the patient was transferred to a specialized cardiovascular institution for possible LVAD implantation. Remarkably, her cardiac function improved, with LVEF recovering to 40%, allowing removal of the Impella device (Day 53). She continues to be managed medically without the need for LVAD implantation, and no recurrence of breast cancer has been observed.

## Discussion

In this case, surgical resection of breast cancer was performed under Impella support to avoid the risk of losing the opportune window for optimal cancer treatment, with the goal of improving long-term prognosis through potential subsequent LVAD implantation or heart transplantation, because histopathological findings confirmed irreversible cardiac damage and uncertain potential for recovery. The key points of the present case are the treatment strategy for severe CTRCD in a cancer patient and the experience of performing breast cancer surgery with Impella support.

The treatment of cancer patients with haemodynamic collapse often presents significant challenges for treatment strategy (*Summary figure*). If cardiac function improves, cancer surgery or additional chemotherapy can be considered after discontinuation of MCS. However, when cardiac recovery is insufficient, patients are considered high risk for chemotherapy or surgery under general anaesthesia. In such cases, proceeding with cancer resection while maintaining Impella support may be a viable strategy to improve long-term outcomes, potentially facilitating future LVAD implantation or heart transplantation. During multidisciplinary discussions, unfavourable outcomes such as poor oncologic prognosis or haemodynamic deterioration were carefully considered. Although we acknowledge that this strategy might carry significant risks, ultimately this therapeutic strategy was selected through consensus among our multidisciplinary team, based on the goal of optimizing the patient’s long-term prognosis.

Guideline-based heart failure therapy is recommended in patients who develop symptomatic severe CTRCD during anthracyclines and HER2 therapy in cardio-oncology guidelines from the European Society of Cardiology.^7,8^ Although LVAD therapy is a treatment option for advanced heart failure, its indication in cancer patients requires careful consideration.^9^ Current guidelines on LVAD recommend against LVAD implantation in patients with a life expectancy of less than 5 years.^10^ A recent study demonstrates that cancer patients with an LVAD generally have poor prognoses.^9^ Moreover, heart transplantation typically requires a cancer-free interval of at least 5 years. Therefore, for young patients with advanced heart failure and active cancer, a treatment strategy focused on achieving cancer remission while maintaining haemodynamic stability using Impella may optimize long-term prognosis.

In the present case, breast cancer surgery was safely performed under Impella support. Despite continuous heparin administration, no perioperative complications including excessive bleeding were observed, underscoring the safety of this approach. These findings highlight the potential role of temporary Impella support in guiding treatment strategies for patients with active cancer and severe decompensated heart failure.^5,6^ Conversely, the potential for increased bleeding and thromboembolic complications, along with haemodynamic instability due to surgical invasiveness, must be taken into account. Such interventions should be undertaken only after careful multidisciplinary discussion of the risks and benefits and when curative cancer resection is expected. Meticulous case selection is therefore imperative.

The use of Impella is time limited, requiring prompt and accurate decision-making regarding the treatment strategy, as the Impella 5.5 has received CE approval for only a maximum of 30 days of support. This case may provide valuable insight into treatment decision-making for cancer patients with severe heart failure, where both oncologic and cardiologic considerations must be balanced under the limited time frame of support devices. Further data are needed to refine the management approach for this complex patient population.

## Lead author biography


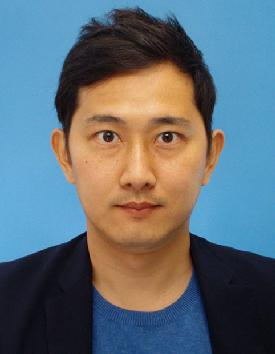


Dr Yoshihito Saijo graduated from the Tokyo Medical University in Tokyo, Japan, in 2011. He completed his specialty in cardiovascular medicine at the faculty of Tokushima University Hospital and is currently a cardiologist at the same institution. Areas of interest included hear failure, valvular disease, cardiotoxicity and cardiomyopathy.

## Supplementary Material

ytaf407_Supplementary_Data

## Data Availability

The data underlying this article are available in the article on reasonable request.

## Notes

**Consent:** The authors confirm that written consent for submission and publication of this case report, including imaging and associated text, has been obtained from the patient in line with COPE guidance.

**Funding:** None declared.
